# Pregnancy outcomes in women with aortic coarctation

**DOI:** 10.1136/heartjnl-2020-317513

**Published:** 2020-10-29

**Authors:** Karishma P Ramlakhan, Daniel Tobler, Matthias Greutmann, Markus Schwerzmann, Lucia Baris, Anji T Yetman, Petros Nihoyannopoulos, Pravin Manga, Eric Boersma, Aldo P Maggioni, Mark R Johnson, Roger Hall, Jolien W Roos-Hesselink, ROPAC investigators group

**Affiliations:** 1 Department of Cardiology, Erasmus MC, Rotterdam, The Netherlands; 2 Department of Cardiology, University Hospital Basel, University of Basel, Basel, Switzerland; 3 Department of Cardiology, University Heart Center, University Hospital Zurich, Zurich, Switzerland; 4 Center for Congenital Heart Disease, University Hospital Inselspital, University of Bern, Bern, Switzerland; 5 Division of Pediatric Cardiology, University of Nebraska Medical Center, Children's Hospital and Medical Center, Omaha, Nebraska, USA; 6 Department of Cardiology, National Heart and Lung Institute, Hammersmith Hospital, London, UK; 7 Division of Cardiology, Department of Internal Medicine, University of Witwatersrand, Johannesburg, South Africa; 8 Department of Clinical Epidemiology, Erasmus MC, Rotterdam, The Netherlands; 9 EURObservational Research Programme, European Society of Cardiology, Sophia Antipolis, France; 10 Maria Cecilia Hospital, GVM Care & Research, Cotignola, Italy; 11 Department of Obstetric Medicine, Imperial College London, Chelsea and Westminster Hospital, London, UK; 12 Department of Cardiology, University of East Anglia, Norwich, UK

**Keywords:** aortic coarctation, pregnancy, congenital heart disease

## Abstract

**Objective:**

Pregnancy in women with aortic coarctation (CoA) has an estimated moderately increased risk (mWHO II–III) of adverse cardiovascular, obstetric or fetal events, but prospective data to validate this risk classification are scarce. We examined pregnancy outcomes and identified associations with adverse outcomes.

**Methods:**

Pregnancies in women with CoA were selected from the worldwide prospective Registry of Pregnancy and Cardiac Disease (ROPAC, n=303 out of 5739), part of the European Society of Cardiology EURObservational Research Programme. The frequency of and associations with major adverse cardiac events (MACE) and hypertensive disorders (pregnancy-induced hypertension, (pre-)eclampsia or haemolysis, elevated liver enzymes and low platelets syndrome) were analysed.

**Results:**

Of 303 pregnancies (mean age 30 years, pregnancy duration 39 weeks), 9.6% involved unrepaired CoA and 27.1% were in women with pre-existing hypertension. No maternal deaths or aortic dissections occurred. MACE occurred in 13 pregnancies (4.3%), of which 10 cases were of heart failure (3.3%). Univariable associations with MACE included prepregnancy clinical signs of heart failure (OR 31.8, 95% CI 6.8 to 147.7), left ventricular ejection fraction <40% (OR 10.4, 95% CI 1.8 to 59.5), New York Heart Association class >1 (OR 11.4, 95% CI 3.6 to 36.3) and cardiac medication use (OR 4.9, 95% CI 1.3 to 18.3). Hypertensive disorders of pregnancy occurred in 16 (5.3%), cardiac medication use being their only predictor (OR 3.2, 95% CI 1.1 to 9.6). Premature births were 9.1%, caesarean section was performed in 49.7% of pregnancies. Of 4 neonatal deaths, 3 were after spontaneous extreme preterm birth.

**Conclusions:**

The ROPAC data show low MACE and hypertensive disorder rates during pregnancy in women with CoA, suggesting pregnancy to be more safe and better tolerated than previously appreciated.

## Introduction

Contemporary cohorts of patients with repaired aortic coarctation (CoA) have good long-term survival but relatively high cardiac complication rates, including re-coarctation in 34%, aortic aneurysms in 18%, hypertension in 32% and 13-fold increased risk for ischaemic stroke as compared with controls without congenital heart disease.^1–4^ Pregnant women with repaired CoA are currently classified as mWHO II–III in the modified WHO classification.^5^ mWHO II–III represents an intermediate risk of maternal mortality and a moderate-to-severe risk of morbidity, corresponding with a cardiac event rate of 10%–19% during pregnancy. Women with unrepaired severe CoA are considered to be mWHO class IV.^5^ Despite these cautionary numbers, the reported rates of complications during pregnancy vary widely.^6–12^ The mWHO classification is based on expert opinion and the previous literature, which consists of several retrospective studies, a retrospective review of a large administrative database (Nationwide Inpatient Sample, USA) and one prospective cohort study of 49 patients with CoA.^6–12^ These studies used different end points and a variable proportion of women with unrepaired CoA were included.^6–12^

Also, surgical and catheter interventions have improved, meaning that historical studies may not reflect current experience. Consequently, there is a need for a large, prospective contemporaneous study with clearly defined inclusion criteria and end points, to allow us to counsel the increasing number of women who are surviving their surgical correction and considering whether to become pregnant. In this manuscript, we examine maternal and fetal outcomes in women with CoA from the worldwide prospective Registry Of Pregnancy And Cardiac Disease (ROPAC), and identify associations with adverse cardiac outcome and hypertensive disorders.

## Methods

The ROPAC is a worldwide prospective registry of pregnancies in women with structural heart disease. It was initiated in 2007 by the European Society of Cardiology as part of the EURObservational Research Programme. Further details on study protocol and study design were published previously.^13^ Informed consent was obtained when required by the local ethics committee of participating centres. Patients were prospectively included from January 2008 to January 2018, in addition to retrospective inclusions from 2007. A total of 5739 pregnancies were included from 138 participating centres in 53 countries.

### Aortic coarctation

From the total ROPAC cohort of 5739 pregnancies, 303 (5%) pregnancies in women with CoA (mean age 30 years) were identified and included in the study. Both repaired and unrepaired CoA were included. Residual CoA was not specified as a variable in the ROPAC case report form and therefore was not available. Aortic dilatation was defined as an aortic diameter >40 mm.

### Data collection and definitions

Data were collected by the local investigator for each participating centre, using patient record files. Baseline patients’ characteristics before pregnancy included age, parity, primary cardiac diagnosis and concomitant cardiac or valvular disease, prior interventions, cardiovascular risk factors, New York Heart Association (NYHA) functional classification, cardiac medication and if available, echocardiographic parameters. The International Monetary Fund classification was used to define a participating country as emerging or advanced.

### Maternal cardiac end points

Cardiac end points were maternal cardiac death, hospitalisation for cardiac reasons, heart failure during pregnancy or within 6 months post partum, atrial fibrillation/flutter, ventricular tachyarrhythmias, endocarditis, thromboembolic events, aortic dissection and acute coronary syndrome. A major adverse cardiac event (MACE) was defined as a composite outcome of these end points, excluding hospital admission.

### Obstetric and fetal end points

Obstetric and fetal end points were pregnancy-induced hypertension, (pre-)eclampsia or haemolysis, elevated liver enzymes and low platelets (HELLP) syndrome, postpartum haemorrhage, (emergency) caesarean section, fetal mortality >24 weeks gestation, neonatal mortality <6 months, intrauterine growth restriction, preterm delivery (<37 weeks gestational age), low Apgar score (<7) and birth weight, which was classified as low birth weight when <2.500 g. Hypertensive disorders of pregnancy were defined as pregnancy-induced hypertension, (pre-)eclampsia or HELLP syndrome and according to the 2018 International Society for the Study of Hypertension in Pregnancy statement.^14^

### Statistical analysis

Baseline characteristics and outcomes were compared between women with prior hypertension and without prior hypertension, as well as between repaired and unrepaired CoA and between women with and without bicuspid aortic valve (BAV). Additionally, the outcomes of MACE and hypertensive disorders were evaluated after dividing the cohort in three risk profiles: (1) no prior hypertension and repaired CoA, (2) prior hypertension or unrepaired CoA and (3) both prior hypertension and unrepaired CoA. Normality of continuous data was examined using QQ-plots and subsequently data are presented as mean and SD, or as median and IQR. Differences between the groups were calculated using Student’s t-tests, or Mann-Whitney U tests for skewed data. Categorical data are presented as percentages and compared using χ^2^ tests.

Univariate logistic regression was used to identify associations with MACE or hypertensive disorders. The number of events (n=13) did not allow for multivariate analysis. Missing data were handled with multiple imputation. A two-sided p value <0.05 was considered to be statistically significant. There were no corrections for multiple testing performed. All analyses and imputations were performed using IBM SPSS Statistics V.25.0 (IBM).

### Patient and public involvement

This research was done without patient involvement.

## Results

Baseline characteristics of the 303 pregnancies in women with CoA are presented in table 1. Pre-existing hypertension was reported in 81 (27.1%) of pregnancies and in 127 (41.9%) cardiac medication was used before pregnancy. Twenty-nine (9.6%) pregnancies were in women with unrepaired CoA. None of the women had Turner’s syndrome or Shone complex.

**Table 1:** Baseline characteristics of the total coarctation cohort, women with pre-existing hypertension and women without pre-existing hypertension

	Total cohort (n=303)	Pre-existing hypertension (n=81)	No pre-existing hypertension (n=222)	P value
Demographics				
Age (mean, SD)	29.9 (5.3)	30.6 (5.4)	29.7 (5.3)	0.349
Nulliparity (%)	155 (51.2)	45 (55.6)	109 (50)	0.393
BMI (median, IQR)	23.3 (21.4–27.4)	24.1 (21.3–27.1)	23.0 (21.5–27.4)	0.953
Emerging country (%)	63 (20.8)	19 (23.5)	43 (19.7)	0.479
Prepregnancy characteristics				
Current smoker (%)	9 (3.6)	3 (4.3)	6 (3.4)	0.735
Diabetes mellitus (%)	6 (2)	0 (0)	5 (2.3)	0.172
Atrial fibrillation (%)	1 (0.3)	0 (0)	1 (0.5)	0.541
Signs of heart failure (%)	8 (2.6)	2 (2.5)	6 (2.8)	0.893
LVEF <40% (%)	7 (2.3)	1 (1.2)	6 (2.8)	0.440
Cardiac medication use before pregnancy (%)	127 (41.9)	60 (74.1)	66 (30.3)	**<0.001**
Beta-blocker	37 (12.2)	22 (27.2)	15 (6.9)	**<0.001**
ACE-inhibitor	9 (3.0)	7 (8.6)	2 (0.9)	**0.001**
Calcium channel blocker	11 (3.6)	10 (12.3)	1 (0.5)	**<0.001**
Diuretics	2 (0.7)	1 (1.2)	1 (0.5)	0.464
NYHA class I (%)	266 (87.8)	67 (82.5)	197 (90.4)	0.067
NYHA class II (%)	31 (10.2)	13 (16)	17 (7.8)	**0.035**
NYHA class III (%)	3 (1)	0 (0)	3 (1.4)	0.289
NYHA class IV (%)	0 (0)	0 (0)	0 (0)	n.a.
Unrepaired coarctation (%)	29 (9.6)	19 (12.3)	18 (8.3)	0.281
Surgery for recoarctation	41 (13.5%)	15 (18.5)	26 (11.9%)	0.141
Bicuspid aortic valve (%)	123 (40.6)	30 (37)	92 (42.2)	0.419
Ventricular septal defect (%)	37 (12.2)	5 (6.2)	32 (14.7)	**0.047**
Persistent arterial duct (%)	17 (5.6)	3 (3.7)	14 (6.4)	0.367
Aortic dilatation >40 mm (%)	11 (3.6)	3 (3.7)	8 (3.7)	0.989
Valve replacement (%)*	15 (5)	3 (3.7)	12 (5.5)	0.526
Aortic stenosis (%)	33 (10.9)	9 (11.1)	24 (11.0)	0.980
Mild	20 (6.6)	4 (4.9)	16 (7.3)	
Moderate	13 (4.3)	5 (6.2)	8 (3.7)	
Severe	0 (0)	0 (0)	0 (0)	
Aortic regurgitation (%)	62 (17.3)	11 (15.6)	41 (18.8)	0.529
Mild	40 (13.3)	8 (9.9)	32 (14.7)	
Moderate	12 (4)	3 (3.7)	9 (4.1)	
Severe	0 (0)	0 (0)	0 (0)	
Mitral stenosis (%)	4 (1.3)	0 (0)	4 (1.8)	0.219
Mitral regurgitation (%)	52 (17.4)	15 (18.5)	36 (16.7)	0.796
Mild	47 (15.7)	13 (16)	33 (15.3)	
Moderate	5 (1.7)	2 (2.5)	3 (1.4)	
Severe	0 (0)	0 (0)	0 (0)	
Echocardiogram data available	160 (53)	–	–	

P values were calculated between the groups pre-existing hypertension and no pre-existing hypertension, using χ^2^ tests and Mann-Whitney U tests where appropriate. Bold values denote statistical significance at the p<0.05 level.

*13 aortic, 1 mitral and 1 tricuspid valve replacement.

BMI, body mass index (kg/m^2^); LVEF, left ventricular ejection fraction; n.a., not available; NYHA, New York Heart Association.

Baseline characteristics of women with unrepaired CoA and women with BAV are described in online supplemental table S1 and S2. Prepregnancy characteristics for unrepaired CoA were comparable to the patients with repaired CoA. Women with BAV more often had prepregnancy aortic dilatation (8.1% vs 0.6%, p=0.001) and aortic valve stenosis (21.1% vs 3.9%, p<0.001), compared with women without BAV.

10.1136/heartjnl-2020-317513.supp1 Supplementary data

Women in emerging countries were more likely to have prepregnancy signs of heart failure (12.7% vs 0%, p<0.001) and to be in NYHA class >1 (25.4% vs 7.5%, p<0.001). They did not have more concomitant cardiac lesions compared with women in high-income countries (68.3% vs 64.2%, p=0.545), but more often had unrepaired CoA (20.6% vs 6.7%, p=0.001).

### Cardiovascular outcomes

No maternal mortality occurred during pregnancy or up to 6 months post partum (table 2). Overall, there were 23 (7.6%) hospital admissions, and 13 (4.3%) of those were due to MACE: heart failure in 10 (3.3%) and supraventricular arrhythmia in 3 (1%) patients. MACE are described in further detail in table 3. Of 10 patients with heart failure, in 6 it was new-onset heart failure and 2 had prepregnancy left ventricular ejection fraction (LVEF) <40%. Only one had no additional cardiac lesions and in one CoA was unrepaired. Mean gestational age at development of heart failure was 25 (±8) weeks, with 5 patients in the second trimester, 5 patients in the third trimester and no postpartum cases. All three cases of supraventricular arrhythmia were in women with BAV. Out of the remaining 10 hospital admissions, 8 patients were admitted for uncontrolled hypertension (without heart failure) and for 2 patients the reason for hospital admission was unknown. There were no peripartum complications, no aortic dissections, no cerebrovascular events and no therapeutic interventions performed during pregnancy.

**Table 2:** Cardiovascular, obstetric and fetal outcome

	Overall cohort (n=303)	Pre-existing hypertension (n=81)	No pre-existing hypertension (n=222)	P value
Cardiovascular outcomes				
Maternal mortality (%)	0 (0)	0 (0)	0 (0)	n.a.
Hospital admission for cardiac reasons (%)	23 (7.6)	12 (14.8)	11 (5)	**0.005**
Heart failure (%)	10 (3.3)	4 (4.9)	6 (2.8)	0.350
Hospital admission for uncontrolled hypertension (%)	8 (2.6)	5 (6.2)	3 (1.4)	**0.022**
Atrial fibrillation or flutter (%)	3 (1)	0 (0)	3 (1.4)	0.289
Ventricular tachyarrhythmias (%)	0 (0)	0 (0)	0 (0)	n.a.
Endocarditis (%)	0 (0)	0 (0)	0 (0)	n.a.
Thromboembolic event (%)	0 (0)	0 (0)	0 (0)	n.a.
Aortic dissection (%)	0 (0)	0 (0)	0 (0)	n.a.
Acute coronary syndrome during pregnancy (%)	0 (0)	0 (0)	0 (0)	n.a.
Overall MACE (%)	13 (4.3)	4 (4.9)	9 (4.1)	0.760
Obstetric and fetal outcome				
Hypertensive disorders (%)	16 (5.3)	6 (7.4)	10 (4.6)	0.336
Pregnancy-induced hypertension	8 (2.6)	0 (0)	8 (3.7)	0.081
Pre-eclampsia	8 (2.6)	6 (7.4)	2 (0.9)	**0.002**
Eclampsia or HELLP	0 (0)	0 (0)	0 (0)	n.a.
Postpartum haemorrhage (%)	5 (1.7)	1 (1.2)	4 (1.8)	0.719
Caesarean section (%)	143 (49.7)	46 (58.2)	96 (46.6)	0.079
Emergency caesarean section	31 (10.2)	13 (16)	18 (8.3)	**0.049**
For cardiac reasons	7 (2.3)	3 (3.7)	4 (1.8)	0.342
Fetal mortality >24 weeks (%)	1 (0.3)	0 (0)	1 (0.3)	0.387
Neonatal mortality (%)	4 (1.3)	1 (1.2)	3 (1.4)	0.925
IUGR (%)	12 (4.0)	6 (7.4)	6 (2.8)	0.068
Preterm delivery (%)	25 (9.1)	7 (9)	18 (9.2)	0.947
Low Apgar score <7 (%)	15 (5)	12 (5.5)	3 (3.7)	0.526
Low birth weight <2.5 kg (%)	20 (6.6)	7 (8.6)	13 (6.0)	0.410
Mean birth weight (SD)	3132.8 (566.1)	3024.2 (602.9)	3168.6 (552.8)	0.131
With beta-blocker (n=56)	3018.8 (415.84)	3116.9 (374.4)	2920.6 (438.2)	0.077
Without beta-blocker (n=133)	3180.9 (613.6)	2887.6 (828.1)	3230.1 (562.6)	**0.024**
	P=0.072*	P=0.204*	P=0.008*	

P values were calculated between the groups pre-existing hypertension and no pre-existing hypertension, using χ^2^ tests and unpaired t-tests where appropriate. Bold values denote statistical significance at the p<0.05 level.

*P values were calculated for birth weight between the groups with and without beta-blocker, using unpaired t-tests.

HELLP, haemolysis, elevated liver enzymes and low platelets syndrome; IUGR, intrauterine growth restriction; MACE, major adverse cardiac event; n.a., not available.

**Table 3:** Major adverse cardiac events in women with aortic coarctation

No	Diagnosis	Comorbidities	Prepregnancy cardiac function	GA at event	Medication and interventions	Timing and mode of delivery
1	HF	VSD; pulmonary hypertension; aortic stenosis	NYHA 3 LVEF >40% Signs of HF: no	16	Sildenafil, bosentan, LMWH	34+2 CS
2	HF	VSD; mitral stenosis	NYHA 1 LVEF <40% Signs of HF: no	23	Metoprolol, furosemide	34+4 CS
3	HF	VSD; pulmonary valve stenosis, aortic regurgitation	NYHA 2 LVEF <40% Signs of HF: no	33	Metoprolol, spironolacton	35+1 CS
4	HF	PDA; BAV; aortic regurgitation	NYHA 1 LVEF >40% signs HF: yes	33	Unknown	36+2 eCS
5	HF	VSD; aortic stenosis and regurgitation; HT	NYHA 2 LVEF >40% Signs of HF: no	28	Unknown	35 CS
6	HF	PDA	NYHA 2 LVEF >40% signs HF: yes	13	Metoprolol	38+4 CS
7	HF	None	NYHA 2 LVEF >40% signs HF: yes	28	Unknown; diagnostic aortography	37+1 eCS
8	HF	Unrepaired CoA; HT	NYHA 1 LVEF >40% Signs of HF: no	38	Unknown	39+5 CS
9	HF	ASD; mitral regurgitation; HT	NYHA 2 LVEF >40% Signs of HF: no	26	Calcium channel blocker, methyldopa	38+5 Vaginal
10	HF	Mitral regurgitation; HT	NYHA 2 LVEF >40% signs HF: yes	16	Unknown	38 Vaginal
11	AF	BAV; mitral regurgitation	NYHA unknown LVEF >40% Signs of HF: no	Unknown	Metoprolol	Unknown Vaginal
12	AF	BAV; aortic stenosis and regurgitation; mitral stenosis and regurgitation; pulmonary valve regurgitation	NYHA 1 LVEF >40% Signs of HF: no	16	Metoprolol, acetylsalicylic acid	39+6 Vaginal
13	AF	BAV; aortic and mitral stenosis	NYHA 1 LVEF >40% Signs of HF: no	29		40+6 Vaginal

AF, atrial fibrillation; BAV, bicuspid aortic valve; CoA, aortic coarctation; (e)CS, (emergency) caesarean section; GA, gestational age (weeks+days if available); HF, heart failure; HT, hypertension; LMWH, low molecular weight heparin; LVEF, left ventricular ejection fraction; NYHA, New York Heart Association.

Women with pre-existing hypertension had more hospital admissions for cardiac reasons compared with women without pre-existing hypertension (14.8% vs 5%, p=0.005), but there was no statistical difference in MACE (4.9% vs 4.1%, p=0.76). Admissions for uncontrolled hypertension were also more frequent than in women without pre-existing hypertension (6.2% vs 1.4%, p=0.022). Women with unrepaired CoA were also hospitalised more often than women with repaired CoA (20.7% vs 6.2%, p=0.005), predominantly for uncontrolled hypertension (13.8% vs 1.5%, p<0.001). There were no further differences in cardiovascular outcome (MACE 3.4% vs 4.4%; p=0.81). Despite aortic dilatation at baseline in 8.1% of women with BAV, there were no dissections and no difference in MACE between women with BAV and those without (3.3% vs 5%, p=0.461).

**Figure 1:**
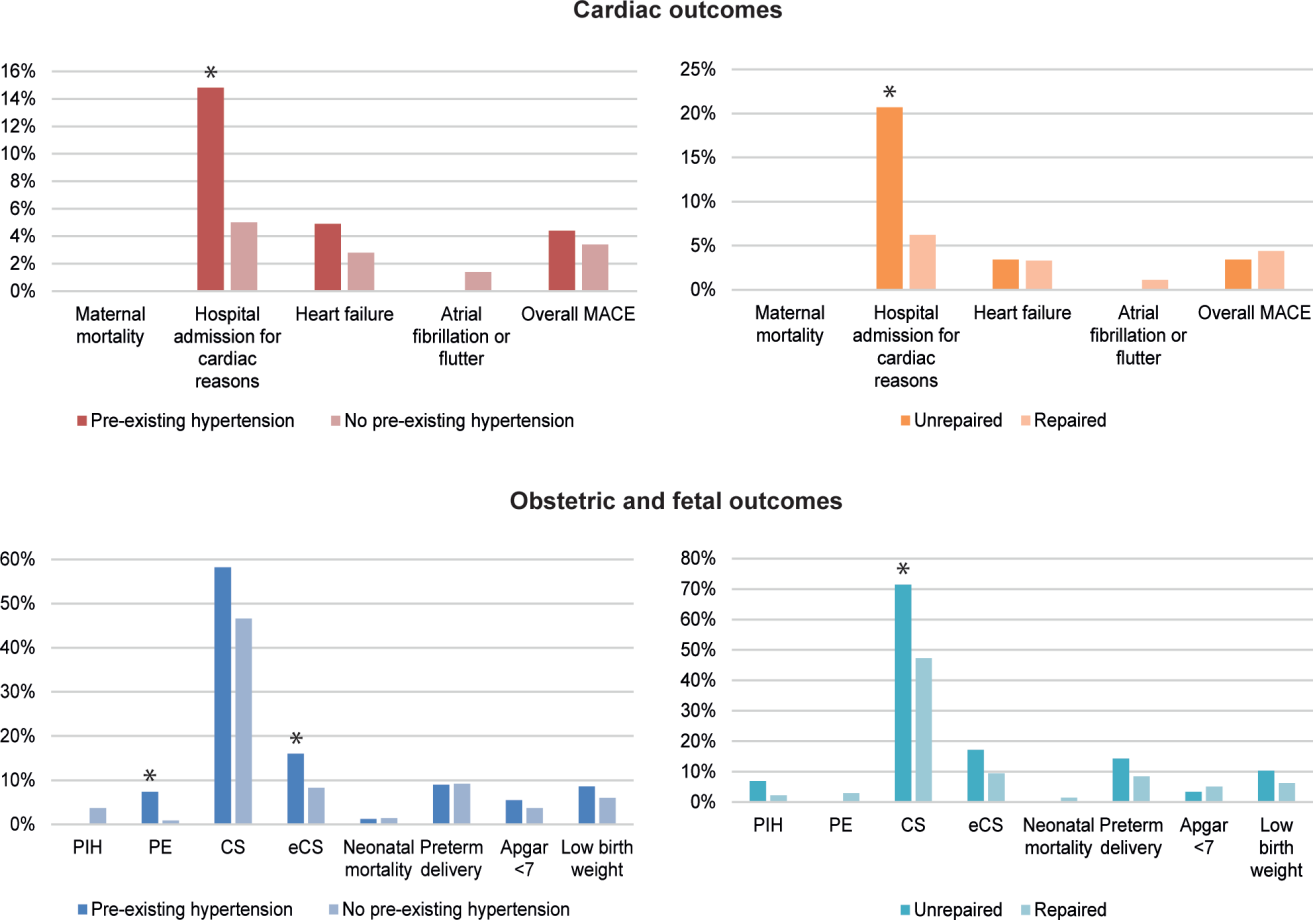
Cardiac events (top) and obstetric and fetal events (bottom) in women with aortic coarctation. Pre-existing hypertension versus no pre-existing hypertension (left) and unrepaired versus repaired (right). *P<0.05. There were no cases of ventricular tachyarrhythmia, endocarditis, thromboembolic events, acute coronary syndrome or aortic dissection. (e)CS, (emergency) caesarean section; MACE, major adverse cardiac event; PE, (pre-)eclampsia; PIH, pregnancy-induced hypertension; PPH, postpartum haemorrhage.

Maternal outcomes are summarised in figure 1 and table 2 (comparison between pre-existing hypertension and no pre-existing hypertension), online supplemental table S3 (comparison between repaired and unrepaired CoA) and online supplemental table S4 (comparison between BAV and no BAV).

### Obstetric and fetal outcomes

Hypertensive disorders of pregnancy were reported in 16 (5.3%) women, as pregnancy-induced hypertension and pre-eclampsia in 8 (2.6%) pregnancies both. There were no cases of eclampsia or HELLP syndrome. Compared with women without pre-existing hypertension, women with pre-existing hypertension developed pre-eclampsia more often (0.9% vs 7.4%, p=0.002).

Caesarean section was performed in 49.7% of the CoA cohort, the majority of which were planned. Emergency caesarean sections were 10.2% and mainly for obstetric reasons, with 2.3% for cardiac reasons. Women with pre-existing hypertension were more likely to have an emergency caesarean section than women without pre-existing hypertension (16% vs 8.3%, p=0.049), but not more often for cardiac reasons. Women with unrepaired CoA underwent caesarean section more often compared with women with repaired CoA (71.4% vs 47.3%; p=0.015), but there was no difference in emergency caesarean sections or emergency caesarean sections for cardiac reasons.

Preterm delivery took place in 25 pregnancies (9.1%) at a median 35 (Q1–Q3 33–36) weeks of gestation, induced in 6% and spontaneous in 3.1%. Intrauterine fetal demise >24 weeks gestational age occurred in one case (0.3%) and neonatal mortality occurred in four cases (1.3%). Three of these deaths were after extremely premature delivery (gestational age between 24 and 26 weeks) and were due to the resulting complications (cerebral haemorrhage; infection). One neonate, born at 38 weeks gestational age, died of an unknown cause. Four (1.3%) newborns were diagnosed with non-cardiac congenital disease and 10 (3.3%) with congenital heart disease: 4 septal defects, 2 aortic abnormalities, 2 univentricular hearts, 1 persistent ductus arteriosus and 1 pulmonary valve stenosis. There were no increased adverse fetal outcomes in the subgroup analyses for women with pre-existing hypertension, women with unrepaired CoA or women with BAV.

Obstetric and fetal outcomes are summarised in figure 1 and table 2 (comparison between pre-existing hypertension and no pre-existing hypertension), online supplemental table S3 (comparison between repaired and unrepaired CoA) and online supplemental table S4 (comparison between BAV and no BAV).

### Univariable associations with adverse outcomes

**Figure 2:**
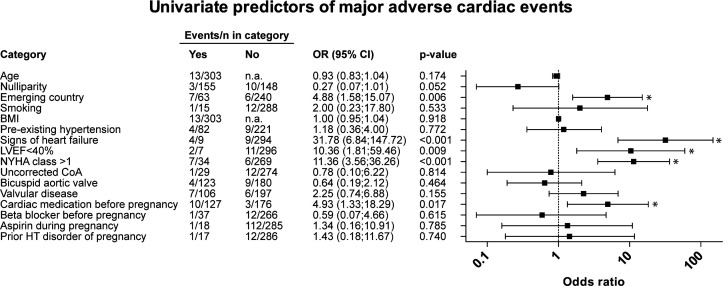
Univariate regression analysis, identifying predictors of major adverse cardiac events in pregnancy. Asterisk (*) denotes statistical significance. BMI, body mass index (kg/m^2^); CoA:, aortic coarctation.; HT, disorder: hypertensive disorder; LVEF, left ventricular ejection fraction; n.a., not available; NYHA, New York Heart Association.

Univariable associations with MACE are displayed in figure 2. In the univariate analysis, emerging country (OR 4.9, 95% CI 1.6 to 15.1), prepregnancy clinical signs of heart failure (OR 31.8, 95% CI 6.8 to 147.7), left ventricular systolic function (LVEF) <40% (OR 10.4, 95% CI 1.8 to 59.5), NYHA class >1 (OR 11.4, 95% CI 3.6 to 36.3) and cardiac medication use before pregnancy (OR 4.9, 95% CI 1.3 to 18.3) were characteristics predicting for MACE. Multivariate analysis was not possible due to the low number of events.

**Figure 3:**
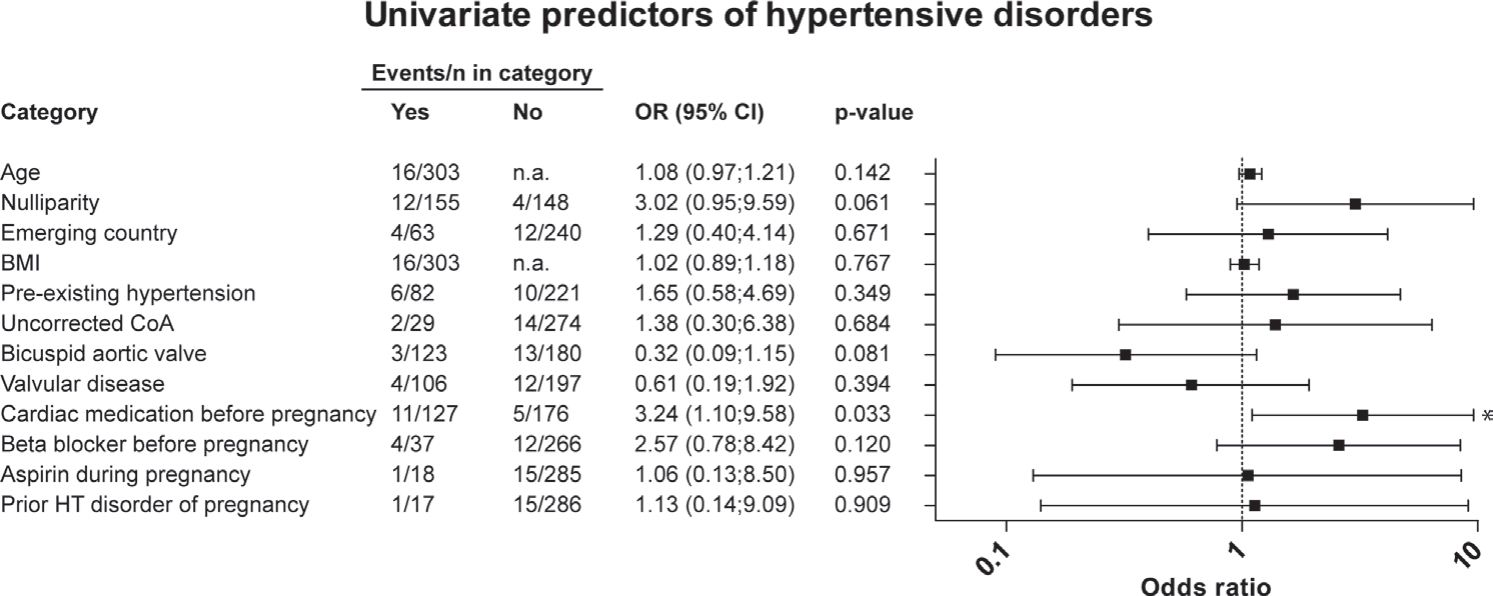
Univariate regression analysis, identifying predictors of hypertensive complications in pregnancy. Asterisk (*) denotes statistical significance. BMI, body mass index (kg/m^2^); CoA, aortic coarctation; HT, hypertensive disorder; LVEF, left ventricular ejection fraction; n.a., not available; NYHA, New York Heart Association.

Univariate analysis for hypertensive disorders is displayed in figure 3. Only prepregnancy cardiac medication use was a statistically significant association (OR 3.2, 95% CI 1.1 to 9.6). Acetylsalicylic acid, commonly used as a prophylaxis in women with risk factors for pre-eclampsia, was used in 5.9% of all pregnancies between 14 and 36 weeks of gestation. It was not associated with a lower incidence of hypertensive disorders (OR 1.1, 95% CI 0.1 to 8.5).

In table 4, MACE and hypertensive disorders were tested against three different CoA profiles, but there were no differences in complication rates between the risk groups. MACE rate in emerging countries was significantly higher than in advanced countries (11.1% vs 2.5%, p=0.003), but no such difference existed for hypertensive disorders (6.3% vs 5%, p=0.670).

**Table 4:** Cardiac and hypertensive complications stratified according to CoA characteristics

	0 n=204	1 n=89	2 n=10	P value
MACE	9 (4.4)	3 (3.4)	1 (10.0)	0.611
Hypertensive disorders	8 (3.9)	8 (9)	0 (0)	0.153

P value was calculated between the risk categories, using a χ^2^ test.

0=no prior hypertension and repaired coarctation; 1=prior hypertension or unrepaired coarctation; 2=prior hypertension and unrepaired coarctation.

CoA, aortic coarctation; MACE, major adverse cardiac event.

## Discussion

**Figure 4:**
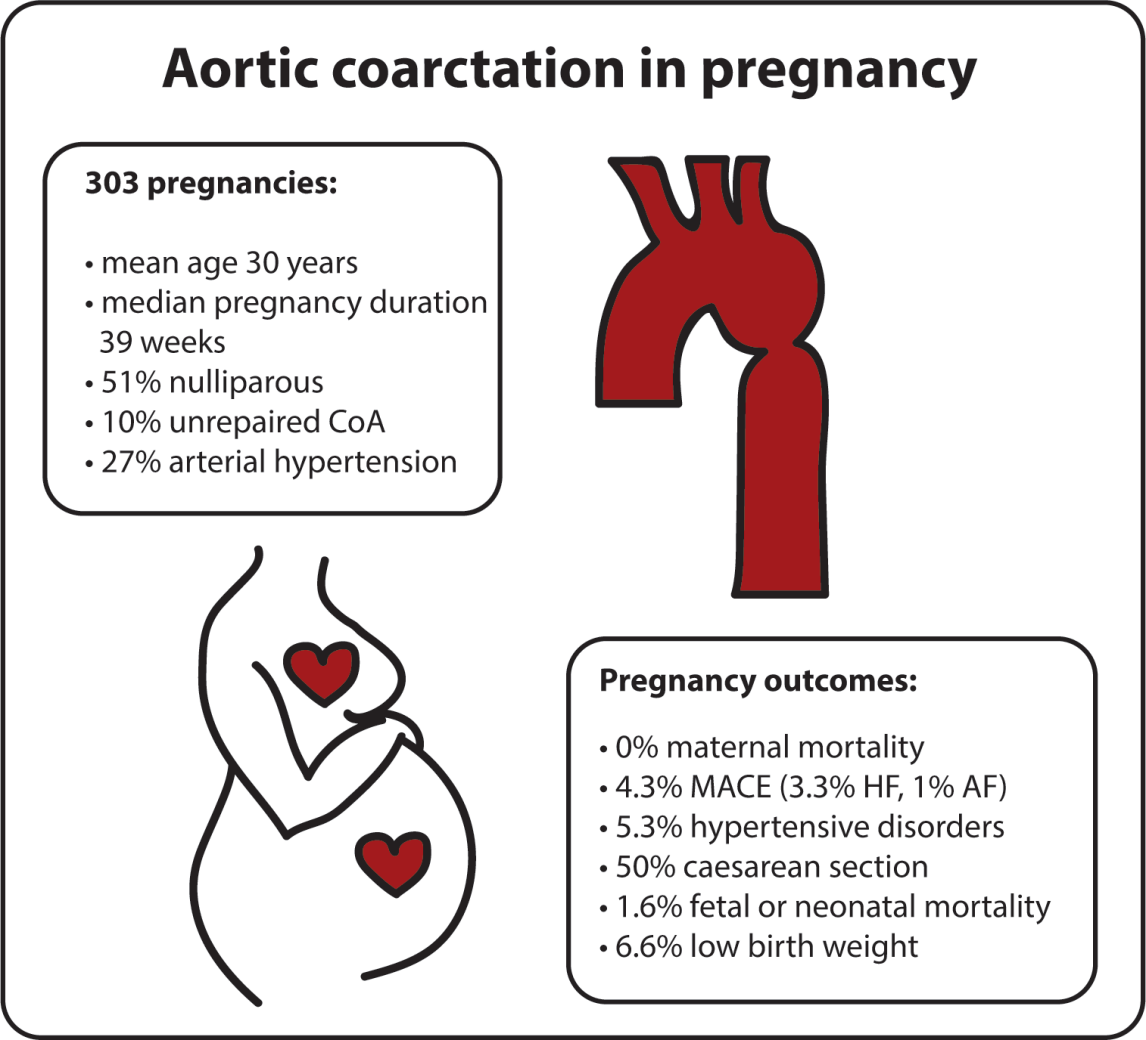
Summarising figure of pregnancy outcomes in women with aortic coarctation. AF, atrial fibrillation; HF, heart failure; MACE, major adverse cardiac event (defined as maternal cardiac death, heart failure, atrial fibrillation or flutter, ventricular tachyarrhythmia, endocarditis, thromboembolic events, aortic dissection and acute coronary syndrome).

Based on 303 pregnancies in women with CoA from an international prospective registry, pregnancy seems to be safe and well-tolerated (figure 4). Cardiac event rates were lower than predicted according to the mWHO class risk stratification score. Another surprising finding was that hypertensive disorders of pregnancy were not more frequent than reported in the general population.

There were no maternal deaths in our cohort. One older report documents a disconcertingly high mortality rate of 9%.^15^ Beauchesne *et al* 9 describes one (1%) fatal dissection in a patient with Turner’s syndrome and twin pregnancy in a cohort of 118 CoA pregnancies. More recent and contemporary studies report no maternal mortality,^6 7 10 11^ which is in line with our results. The largest cohort to date (n=697) from Krieger *et al* did not report a mortality rate, but instead includes maternal death in their composite adverse cardiovascular outcome (5%).^12^

The MACE rate of 4.3% is lower than the predicted 10%–19% event rate for patients with a mWHO class II–III risk score.^5^ In particular, no cerebrovascular events nor aortic dissections occurred, which are recognised generally as the most feared complications in young adult patients with CoA,^11 16^ with pregnancy as a particular period of risk due to hormonally mediated vessel wall weakness and increased thrombogenicity.^17 18^ The MACE rates in this study are in line with recent literature. Krieger *et al* 12 reports a 5% MACE rate, which is a similar event rate to our study. In a prospective observational cohort study of 49 pregnancies, one major cardiovascular event (2%) occurred^6^ and three other retrospective studies report zero major cardiac events.^7 10 11^ In our study, predictors of cardiac events were prepregnancy signs of heart failure, LVEF <40%, NYHA class >1 and cardiac medication use before pregnancy, along with living in an emerging country. Higher rates of adverse outcomes in women from emerging countries are likely explained by a higher rate of prepregnancy cardiac morbidity (higher rate of prepregnancy heart failure and higher functional class) and higher rates of unrepaired CoA. It is important to further evaluate specific risk factors determining pregnancy outcomes in women from emerging countries in order to elaborate strategies to improve these outcomes. Generally, our data indicate a profile of women with heart failure as the group most at risk for developing MACE. Considering the findings reported here and other recent literature, mWHO class II instead of II–III seems a more appropriate risk assessment for pregnancies in asymptomatic women with CoA without signs of heart failure and/or normal or mildly reduced LVEF. Beside MACE, hospital admissions were most often because of uncontrolled hypertension, in particular in women with pre-existing hypertension or unrepaired CoA. Strict blood pressure control seems advisable in these pregnancies.

We found a surprisingly low number of hypertensive disorders of pregnancy (5.3%) and comparable (low) rates of pre-eclampsia compared with the general population.^19 20^ These findings are in contrast to the previous studies, where hypertensive disorders have been found as the most frequent complication of pregnancy in women with repaired CoA. Hypertensive disorder rates were reported between 14% and 20%,^7 10 12^ up to 30% if pre-existing hypertension is included in the definition of hypertensive outcomes.^9 11 12^ These differences may thus be partially explained by heterogeneous definitions of hypertensive disorders during pregnancy. Given the focus on systemic hypertension in patients with repaired or unrepaired CoA, one may also speculate that the identification and treatment of pre-existing hypertension in women with CoA compared with unselected pregnancy cohorts may have led to this surprisingly low number of hypertensive disorders of pregnancy. In this regard, it may be noteworthy the only previous prospective study found no difference in hypertensive disorder rates between women with CoA and healthy controls.^6^ In the study by Siegmund *et al*,^6^ pregnancy-induced hypertension was found in 18% of pregnancies and pre-eclampsia in 2% of pregnancies.

Pregnancy in unrepaired CoA is underreported and caregivers might be apprehensive about complications in this population, as demonstrated by the high caesarean section rate (71.4%). However, we did not find any increase in cardiac, obstetric or fetal adverse outcomes when compared with the repaired group. In the only study to describe pregnancy outcomes in unrepaired CoA, no significant differences between the repaired and unrepaired group were found.^9^ In our cohort of women with unrepaired CoA, the severity of the coarctation was unknown and therefore our outcomes may not be applicable to women with severe unrepaired CoA.

The caesarean section rate of 49.7% is markedly higher than the globally observed rate of 21% and the recommended rate of 10%–15%,^21 22^ but similar to the 47% found in the total ROPAC cohort of all women with structural cardiac disease.^23^ In unrepaired CoA, the caesarean section rate is even more pronounced (71%). This is not according to current guidelines on pregnancy in cardiac disease, which state that vaginal delivery is almost always the preferred option—excluding a few severe diseases, which do not include CoA.^5^ Planned caesarean sections in women with cardiac disease such as CoA offer no maternal advantage while they increase adverse fetal outcome.^5 24^

We also found a surprisingly low rate (3.3%) of congenital heart disease in the offspring, considering that the recurrence risk is 4%–6.5% for non-syndromic maternal CoA and higher for syndromic CoA.^25^ One may speculate that isolated bicuspid valves in the offspring has been underdiagnosed, as there was no systematic echocardiographic screening of the neonates. The negative effect of beta-blockers on fetal birth weight did not reach statistical significance in our cohort, despite clear evidence for this association in both the general population and in women with cardiac disease.^26–28^

### Limitations

This study has some limitations due to the nature of the observational data. The ROPAC provides data on individual pregnancies and does not allow identification of the number of women having multiple pregnancies. As the ROPAC was designed to be applicable for all types of structural heart disease, CoA-specific parameters such as peak gradients across the coarctation or the prevalence of recoarctation were not included. The registry includes no specification for indication of prepregnancy medication. Particularly in unrepaired CoA or in women with re-coarctation, information on the severity of the coarctation would be of importance for the generalisability of the results. Notable is the limited prevalence of pre-existing hypertension or antihypertensive medication use in the unrepaired CoA group, which suggests that this cohort mainly involves mild unrepaired CoA or pseudocoarctation. Clinical details such as blood pressure difference between upper and lower extremities or variables of heart failure such as biomarkers were also not available. Although the sample size of this study is large compared with the literature, the number of events is low, which prevented a multivariate analysis of risk factors and should be considered in the interpretation of the study results including the univariate analyses.

## Conclusion

In this prospective observational study of 303 pregnancies in women with aortic coarctation, MACE were rare and no maternal deaths or aortic dissections occurred. Univariable associations with MACE are prepregnancy clinical signs of heart failure, LVEF <40%, NYHA class >1, cardiac medication use before pregnancy and hailing from an emerging country. Hypertensive disorders of pregnancy are not more prevalent than in the general population. Although only a limited amount of data are available with few end points, these findings suggest pregnancy in women with CoA to be more safe and better tolerated than previously appreciated.

**Key questions**

**What is already known on this subject?**

- Aortic coarctation is a common congenital heart defect and considered a high-risk condition.
- Pregnancy is currently classified as modified WHO risk class II–III, which corresponds to an expected maternal cardiac event rate of 10%–19%, and hypertensive disorders of pregnancy are reported in 13%–30%; however, pregnancy outcomes have only been studied in small retrospective cohorts and clinical guidelines are mainly based on expert opinion.

**What might this study add?**

- This study of 303 pregnant women with aortic coarctation is based on data from a prospective registry and exceeds the numbers of patients in previous studies, making it an important contribution to the current literature.
- We found pregnancy to be better tolerated than previously thought, with a maternal cardiac event rate of 4.3% and hypertensive disorders of pregnancy occurring in 5.3%, the latter does not exceed the average in the general population.
- Even women with unrepaired coarctation do not have increased adverse cardiac, obstetric and fetal outcomes.

**How might this impact on clinical practice?**

- The data from this prospective observational study on pregnancies in women with aortic coarctation show that major adverse cardiac events are rare and hypertensive disorders of pregnancy are not more prevalent than in the general population.
- This suggests that women with aortic coarctation without risk factors can safely embark on pregnancy and should be counselled accordingly.
