# Supplementary material for: Pregnancy outcomes in women with aortic coarctation
Source: Heart. 2020 Oct 29;107(4):290–8. doi: 10.1136/heartjnl-2020-317513 (PMC7873427; doi:10.1136/heartjnl-2020-317513)
Supplement: Supplementary data [file heartjnl-2020-317513supp001.pdf]

SUPPLEMENTARY FILE

Supplementary table S1 Baseline characteristics of women with repaired coarctation versus women with unrepaired coarctation

	Total cohort (n=303)	Repaired CoA (n=274)	Unrepaired CoA (n=29)	P-value
**Demographics**				
Age (mean, SD)	29.9 (5.3)	29.9 (5.4)	29.7 (4.8)	0.311
Nulliparity (%)	155 (51.2)	139 (50.7)	16 (55.2)	0.649
BMI (median, IQR)	23.3 (21.4-27.4)	23.4 (21.5-27.4)	22.3 (19.9-25.7)	0.177
Emerging country (%)	63 (20.8)	50 (18.2)	13 (44.8)	**0.001**
**Pre-pregnancy characteristics**				
Current smoker (%)	9 (3.6)	8 (3.6)	1 (4)	0.917
Hypertension (%)	81 (27.1)	71 (26.2)	10 (35.7)	0.281
Diabetes mellitus (%)	6 (2)	6 (2.3)	0 (0)	0.413
Atrial fibrillation (%)	1 (0.3)	1 (0.4)	0 (0)	0.745
Signs of heart failure (%)	8 (2.6)	7 (2.6)	1 (3.4)	0.778
LVEF <40% (%)	7 (2.3)	6 (2.2)	1 (3.4)	0.668
Cardiac medication use before pregnancy (%)	127 (41.9)	115 (42)	12 (41.4)	0.951
Beta blocker	37 (12.2)	37 (13.5)	0 (0)	**0.035**
ACE-inhibitor	9 (3.0)	8 (2.9)	1 (3.4)	0.873
Calcium channel blocker	2 (0.7)	11 (4.0)	0 (0)	0.272
Diuretics	11 (3.6)	2 (0.7)	0 (0.0)	0.644
NYHA class I (%)	266 (87.8)	243 (88.7)	23 (79.3)	0.143
NYHA class II (%)	31 (10.2)	25 (9.1)	6 (20.7)	0.051

Ramlakhan KP, *et al. Heart* 2021; 107:290–298. doi: 10.1136/heartjnl-2020-317513

NYHA class III (%)	3 (1)	3 (1.1)	0 (0)	0.571
NYHA class IV (%)	0 (0)	0 (0)	0 (0)	n.a.
Bicuspid aortic valve (%)	123 (40.6)	114 (41.6)	9 (31)	0.270
Ventricular septal defect (%)	37 (12.2)	35 (12.8)	2 (6.9)	0.358
Persistent arterial duct (%)	17 (5.6)	16 (5.8)	1 (3.4)	0.595
Aortic dilatation >40mm (%)	11 (3.6)	11 (4.0)	0 (0)	0.272
Valve replacement (%)	15 (5)	15 (5.5)	0 (0)	0.196
Aortic stenosis (%)	33 (10.9)	29 (10.6)	4 (13.8)	0.598
Mild	20 (6.6)	17 (6.2)	3 (10.3)	
Moderate	13 (4.3)	12 (4.4)	1 (3.4)	
Severe	0 (0)	0 (0)	0 (0)	
Aortic regurgitation (%)	62 (17.3)	49 (18)	3 (10.3)	0.201
Mild	40 (13.3)	39 (14.3)	1 (3.4)	
Moderate	12 (4)	10 (3.7)	2 (6.9)	
Severe	0 (0)	0 (0)	0 (0)	
Mitral stenosis (%)	4 (1.3)	4 (1.5)	0 (0)	0.511
Mitral regurgitation (%)	52 (17.4)	44 (16.2)	8 (27.6)	**0.045**
Mild	47 (15.7)	41 (15.1)	6 (20.7)	
Moderate	5 (1.7)	3 (1.1)	2 (6.9)	
Severe	0 (0)	0 (0)	0 (0)	

P-values were calculated between the groups repaired and unrepaired coarctation, using chi-square tests and Mann-Whitney tests where appropriate. CoA: aortic coarctation. IQR: inter-quartile range. BMI: body mass index, in kg/m2. LVEF: left ventricular ejection fraction. ACE-inhibitor: angiontensin-converting enzyme inhibitor. NYHA class: New York Heart Association Functional Classification.

Supplementary table S2 Baseline characteristics of women with bicuspid aortic valve versus women without bicuspid aortic valve.

	**Total cohort (n=303)**	**BAV (n=123)**	**No BAV (n=180)**	**P-value**
**Demographics**				
Age (median, IQR)	30.4 (26.4-33.8)	30.3 (25.8-33.9)	30.4 (26.6-33.8)	0.709
Nulliparity (%)	155 (51.2)	62 (50.4)	93 (51.7)	0.829
BMI (median, IQR)	23.3 (21.4-27.4)	23.4 (21.5-27.8)	23.1 (21.2-27.3)	0.395
Emerging country (%)	63 (20.8)	28 (22.8)	35 (19.4)	0.484
**Pre-pregnancy characteristics**				
Current smoker (%)	9 (3.6)	4 (3.9)	5 (3.4)	0.837
Hypertension (%)	81 (27.1)	30 (24.6)	51 (28.8)	0.419
Diabetes mellitus (%)	6 (2)	2 (1.7)	4 (2.3)	0.719
Atrial fibrillation (%)	1 (0.3)	0 (0)	1 (0.8)	0.226
Signs of heart failure (%)	8 (2.6)	3 (2.4)	5 (2.8)	0.851
LVEF <40% (%)	7 (2.3)	1 (0.8)	6 (3.3)	0.152
Cardiac medication use before pregnancy (%)	127 (41.9)	53 (43.1)	74 (41.1)	0.732
Beta blocker	33 (10.9)	20 (11.1)	13 (10.6)	0.882
ACE-inhibitor	4 (1.3)	4 (3.3)	0 (0.0)	**0.015**
Diuretics	2 (0.7)	2 (1.6)	0 (0.0)	0.086
Antiplatelet therapy	11 (3.6)	8 (6.5)	3 (1.7)	**0.027**
Vitamin K antagonist	2 (0.7)	1 (0.8)	1 (0.8)	0.786
NYHA class I (%)	266 (87.8)	108 (87.8)	158 (87.8)	0.994
NYHA class II (%)	31 (10.2)	11 (8.9)	20 (11.1)	0.541
NYHA class III (%)	3 (1)	2 (1.6)	1 (0.6)	0.355
NYHA class IV (%)	0 (0)	0 (0)	0 (0)	n.a.

Ramlakhan KP, *et al. Heart* 2021; 107:290–298. doi: 10.1136/heartjnl-2020-317513

Unrepaired coarctation (%)	29 (9.6)	9 (7.3)	20 (11.1)	0.270
Ventricular septal defect (%)	37 (12.2)	11 (8.9)	26 (14.4)	0.151
Persistent arterial duct (%)	17 (5.6)	6 (4.9)	11 (6.1)	0.647
Aortic dilatation >40mm (%)	11 (3.6)	10 (8.1)	1 (0.6)	**0.001**
Valve replacement (%)	15 (5)	8 (6.5)	7 (3.9)	0.303
Aortic stenosis (%)	33 (10.9)	26 (21.1)	7 (3.9)	**<0.001**
Mild	20 (6.6)	15 (12.2)	5 (2.8)	
Moderate	13 (4.3)	11 (8.9)	2 (1.1)	
Severe	0 (0)	0 (0)	0 (0)	
Aortic regurgitation (%)	62 (17.3)	33 (26.9)	19 (10.7)	**0.001**
Mild	40 (13.3)	27 (22)	13 (7.3)	
Moderate	12 (4)	6 (4.9)	6 (3.4)	
Severe	0 (0)	0 (0)	0 (0)	
Mitral stenosis (%)	4 (1.3)	3 (2.4)	1 (0.6)	0.162
Mitral regurgitation (%)	52 (17.4)	27 (22.1)	25 (14)	0.060
Mild	47 (15.7)	26 (21.3)	21 (11.8)	
Moderate	5 (1.7)	1 (0.8)	4 (2.2)	
Severe	0 (0)	0 (0)	0 (0)	

P-values were calculated between the groups bicuspid aortic valve and no bicuspid aortic valve, using chi-square tests and Mann-Whitney tests where appropriate. BAV: bicuspid aortic valve. IQR: inter-quartile range. BMI: body mass index, in kg/m2. LVEF: left ventricular ejection fraction. ACE-inhibitor: angiontensin-converting enzyme inhibitor. NYHA class: New York Heart Association Functional Classification.

Ramlakhan KP, *et al. Heart* 2021; 107:290–298. doi: 10.1136/heartjnl-2020-317513

Supplementary table S3 Cardiovascular, obstetric and fetal outcome of women with repaired coarctation versus women with unrepaired coarctation.

	Total cohort (n=303)	Repaired CoA (n=274)	Unrepaired CoA (n=29)	P-value
**Cardiovascular outcomes**				
Maternal mortality (%)	0 (0)	0 (0)	0 (0)	n.a.
Hospital admission for cardiac reasons (%)	23 (7.6)	17 (6.2)	6 (20.7)	**0.005**
Heart failure (%)	10 (3.3)	9 (3.3)	1 (3.4)	0.963
Hospital admission for uncontrolled hypertension (%)	8 (2.6)	4 (1.5)	4 (13.8)	**0.000**
Atrial fibrillation or flutter (%)	3 (1)	3 (1.1)	0 (0)	0.571
Ventricular tachyarrhythmias (%)	0 (0)	0 (0)	0 (0)	n.a.
Endocarditis (%)	0 (0)	0 (0)	0 (0)	n.a.
Thromboembolic event (%)	0 (0)	0 (0)	0 (0)	n.a.
Aortic dissection (%)	0 (0)	0 (0)	0 (0)	n.a.
Acute coronary syndrome during pregnancy (%)	0 (0)	0 (0)	0 (0)	n.a.
Overall MACE (%)	13 (4.3)	12 (4.4)	1 (3.4)	0.814
**Obstetric and fetal outcome**				
Hypertensive disorders (%)	16 (5.3)	14 (5.1)	2 (12.5)	0.682
Pregnancy-induced hypertension	8 (2.6)	6 (2.2)	2 (6.9)	0.133
Preeclampsia	8 (2.6)	8 (2.9)	0 (0.0)	0.351
Eclampsia or HELLP	0 (0)	0 (0)	0 (0)	n.a.
Post-partum hemorrhage (%)	5 (1.7)	5 (1.8)	0 (0.0)	0.463
Caesarean section (%)	143 (49.7)	123 (47.3)	20 (71.4)	**0.015**

Ramlakhan KP, *et al. Heart* 2021; 107:290–298. doi: 10.1136/heartjnl-2020-317513

Emergency Caesarean section	31 (10.2)	26 (9.5)	5 (17.2)	0.190
For cardiac reasons	7 (2.3)	6 (2.2)	1 (3.4)	0.668
Fetal mortality >24wks (%)	1 (0.3)	1 (0.4)	0 (0.0)	0.745
Neonatal mortality (%)	4 (1.3)	4 (1.5)	0 (0.0)	0.512
IUGR (%)	12 (4.0)	10 (3.5)	2 (6.9)	0.394
Preterm delivery (%)	25 (9.1)	21 (8.5)	4 (14.3)	0.313
Low apgar score <7 (%)	15 (5)	14 (5.1)	1 (3.4)	0.695
Low birth weight <2.5kg (%)	20 (6.6)	17 (6.2)	3 (10.3)	0.393
Mean birth weight (SD)	3155.1 (514.4)	3139.1 (562.4)	3073.1 (613.1)	0.639

P-values were calculated between the groups repaired and unrepaired coarctation, using chi-square tests and unpaired t-tests where appropriate. CoA: aortic coarctation. MACE: major adverse cardiac event. HELLP: hemolysis, elevated liver enzymes and low platelets syndrome. IUGR: intrauterine growth restriction.

Ramlakhan KP, *et al. Heart* 2021; 107:290–298. doi: 10.1136/heartjnl-2020-317513

Supplementary table S4 Cardiovascular, obstetric and fetal outcome of women with bicuspid aortic valve versus women without bicuspid aortic valve.

	**Overall cohort (n=303)**	**BAV (n=123)**	**No BAV (n=180)**	**P-value**
**Cardiovascular outcome**				
Maternal mortality (%)	0 (0)	0 (0)	0 (0)	n.a.
Hospital admission for cardiac reasons (%)	23 (7.6)	7 (5.7)	16 (8.9)	0.302
Heart failure (%)	10 (3.3)	1 (0.8)	9 (5.0)	**0.045**
Hospital admission for uncontrolled hypertension (%)	8 (2.6)	2 (1.6)	6 (3.3)	0.363
Atrial fibrillation or flutter (%)	3 (1)	3 (2.4)	0 (0)	**0.035**
Ventricular tachyarrhythmias (%)	0 (0)	0 (0)	0 (0)	n.a.
Endocarditis (%)	0 (0)	0 (0)	0 (0)	n.a.
Thromboembolic event (%)	0 (0)	0 (0)	0 (0)	n.a.
Aortic dissection (%)	0 (0)	0 (0)	0 (0)	n.a.
Acute coronary syndrome during pregnancy (%)	0 (0)	0 (0)	0 (0)	n.a.
Overall MACE (%)	13 (4.3)	4 (3.3)	9 (5.0)	0.461
**Obstetric and fetal outcome**				
Hypertensive disorders (%)	16 (5.3)	3 (2.4)	13 (7.2)	0.068
Pregnancy-induced hypertension	8 (2.6)	2 (1.6)	6 (3.3)	0.363
Preeclampsia	8 (2.6)	1 (0.8)	7 (3.9)	0.101
Eclampsia or HELLP	0 (0)	0 (0)	0 (0)	n.a.
Post-partum hemorrhage (%)	5 (1.7)	3 (2.4)	2 (1.1)	0.373
Caesarean section (%)	143 (49.7)	59 (49.6)	84 (49.7)	0.983

Ramlakhan KP, *et al. Heart* 2021; 107:290–298. doi: 10.1136/heartjnl-2020-317513

Emergency Caesarean section	31 (10.2)	14 (11.4)	17 (9.4)	0.585
For cardiac reasons	7 (2.3)	3 (2.4)	4 (2.2)	0.902
Fetal mortality >24wks (%)	1 (0.3)	1 (0.8)	0 (0)	0.226
Neonatal mortality (%)	4 (1.3)	0 (0)	4 (2.2)	0.096
IUGR (%)	12 (4.0)	3 (2.4)	9 (5.0)	0.262
Preterm delivery (%)	25 (9.1)	8 (7.0)	17 (10.6)	0.297
Low apgar score <7 (%)	15 (5)	8 (6.5)	7 (3.9)	0.303
Low birth weight <2.5kg (%)	20 (6.6)	8 (6.5)	12 (6.7)	0.955
Mean birth weight (SD)	3132.8 (566.1)	3145.6 (502.9)	31220 (617.1)	0.776

P-values were calculated between the groups bicuspid aortic valve and no bicuspid aortic valve, using chi-square tests and unpaired t-tests where appropriate. BAV: bicuspid aortic valve. MACE: major adverse cardiac event. HELLP: hemolysis, elevated liver enzymes and low platelets syndrome. IUGR: intrauterine growth restriction.

Ramlakhan KP, *et al. Heart* 2021; 107:290–298. doi: 10.1136/heartjnl-2020-317513

Supplementary table S5 Type of coarctation correction at time of the pregnancy

	**n (%)**
Type known	59 (19.5%)
Of which	
End-to-end anastomosis	16 (27.1%)
Stent	16 (27.1%)
Balloon angioplasty	11 (18.6%)
Subclavian flap	9 (15.3%)
Dacron patch	7 (11.9%)

Appendix

EORP Oversight Committee

Christopher Peter Gale, Chair, GB, Branko Beleslin, RS, Andrzej Budaj, PL, Ovidiu Chioncel, RO, Nikolaos Dagres, DE, Nicolas Danchin, FR, David Erlinge, SE, Jonathan Emberson, GB, Michael Glikson, IL, Alastair Gray, GB, Meral Kayikcioglu, TR, Aldo Maggioni, IT, Klaudia Vivien Nagy, HU, Aleksandr Nedoshivin, RU, Anna-Sonia Petronio, IT, Jolien Roos-Hesselink, NL, Lars Wallentin, SE, Uwe Zeymer, DE.

Executive Committee

Roger Hall GB (Co-Chair), Jolien Roos-Hesselink NL (Co-Chair), Joerg Stein, AT, William Anthony Parsonage, AU, Werner Budts, BE, Julie De Backer, BE, Jasmin Grewal, CA, Ariane Marelli, CA, Harald Kaemmerer, DE, Guillaume Jondeau, FR, Mark Johnson, GB, Aldo P. Maggioni, IT, Luigi Tavazzi, IT, Ulf Thilen, SE, Uri Elkayam, US, Catherine Otto, US, Karen Sliwa, ZA.

ROPAC Investigators

ARGENTINA - Buenos Aires: A. Aquieri, A. Saad, H. Ruda Vega, J. Hojman, J.M. Caparros, M. Vazquez Blanco **AUSTRALIA** - Elizabeth Vale: M. Arstall, C.M. Chung, G. Mahadavan, E. Aldridge, M. Wittwer, Y.Y. Chow, Herston: W.A. Parsonage, K. Lust, New Lambton Heights: N. Collins, G. Warner, R. Hatton, A. Gordon, E. Nyman **AUSTRIA** - Innsbruck: J. Stein , E. Donhauser , Vienna: H. Gabriel **AZERBAIJAN** - Baku: A. Bahshaliyev, F. Guliyev, I. Hasanova, T. Jahangirov, Z. Gasimov **BANGLADESH** - Dhaka: A. Salim, C.M. Ahmed, F. Begum, M.H. Hoque, M. Mahmood, M.N. Islam, P.P. Haque, S.K. Banerjee, T. Parveen **BELGIUM** - Brussels: M. Morissens, Gent: J. De Backer, L. Demulier, M. de Hosson, Leuven: W. Budts, M. Beckx **BOSNIA AND HERZEGOVINA** - Banja

Luka: M. Kozic, M. Lovric, T. Kovacevic-Preradovic **BULGARIA** - Sofia: N. Chilingirova, P. Kratunkov **CANADA** - Edmonton: N. Wahab, S. McLean, Hamilton, Ontario: E. Gordon, L. Walter, Montreal: A. Marelli, A. R. Montesclaros **COLOMBIA** - Medellin: G. Monsalve, C. Rodriguez, F. Balthazar, V. Quintero, W. Palacio, L.A. Mejía Cadavid, E. Munoz Ortiz, F. Fortich Hoyos, E. Arevalo Guerrero, J. Gandara Ricardo, J. Velasquez Penagos **CZECH REPUBLIC** - Hradec Kralove: Z. Vavera, Prague: J. Popelova **DENMARK** - Copenhagen: N. Vejlstrup, L. Grønbeck, M. Johansen, A. Ersboll **EGYPT** - Alexandria: Y. Elrakshy, Assiut: K. Eltamawy , M. Gamal Abd-El Aziz, Benha : A. El Nagar, H. Ebaid, H. Abo Elenin, M. Saed, S. Farag, W. Makled, Cairo: K. Sorour, Z. Ashour, G. El-Sayed, M. Abdel Meguid Mahdy, Minia: N. Taha, A. Dardeer, M. Shabaan, Zagazig: A. Saad, M. Ali **FRANCE** - Nice: P. Moceri, Paris: G. Duthoit, M. Gouton, J. Nizard, L. Baris, S. Cohen, M. Ladouceur, D. Khimoud, B. Iung **GERMANY** - Berlin: F. Berger, A. Olsson, Bonn: U. Gembruch, W.M. Merz, E. Reinert, S. Clade, Y. Kliesch, Essen: C. Wald, Hamburg: C. Sinning, R. Kozlik-Feldmann, S. Blankenberg, E. Zengin-Sahm, G. Mueller, M. Hillebrand, P. Hauck, Y. von Kodolitsch, N. Zarniko, Muenster: H. Baumgartner, R. Schmidt, A. Hellige, Munich: O. Tutarel, H. Kaemmerer, B. Kuschel, N. Nagdyman, Oldenburg: R. Motz **GEORGIA** - Tbilisi: D. Maisuradze **GREECE** - Athens: A. Frogoudaki, E. Iliodromitis, M. Anastasiou-Nana, Marousi, D. Triantafyllis, G. Bekiaris, Thessaloniki: H. Karvounis, G. Giannakoulas, D. Ntiloudi, S.A. Mouratoglou **HUNGARY** - Budapest: A. Temesvari, H. Balint, D. Kohalmi, B. Merkely, C. Liptai, Szeged: A. Nemes, T. Forster, A. Kalapos, K. Berek, K. Havasi, N. Ambrus **INDIA** - Karad: A. Shelke, R. Kawade, S. Patil **INDONESIA** - Bandung: E. Martanto, T.M. Aprami, A. Purnomowati, C.J. Cool, M. Hasan, R. Akbar, S. Hidayat, T.I. Dewi, W. Permadi, D.A. Soedarsono **IRAN** - Tehran: M.M. Ansari-Ramandi, N. Samiei, A. Tabib, F. Kashfi, S. Ansari-Ramandi, S. Rezaei **IRAQ** - Baghdad: H. Ali Farhan, A. Al-Hussein, G. Al-Saedi, G. Mahmood, I.F. Yaseen, L. Al-

Yousuf, M. AlBayati, S. Mahmood, S. Raheem, T. AlHaidari, Z. Dakhil **IRELAND** - Dublin: P. Thornton, J. Donnelly, M. Bowen **ISRAEL** - Beer Yakov: A. Blatt, G. Elbaz-Greener, Hadera: A. Shotan, Haifa: S. Yalonetsky, Rehovot: S. Goland, M. Biener **ITALY** - Bologna: G. Egidy Assenza, M. Bonvicini, A. Donti, A. Bulgarelli, D. Prandstraller, Bolzano: C. Romeo, R. Crepaz, Brescia: E. Sciatti, M. Metra, R. Orabona, Massa: L. Ait Ali, P. Festa, Milan: V. Fesslova , C. Bonanomi, M. Calcagnino, F. Lombardi, A.M. Colli, M.W. Ossola, C. Gobbi, E. Gherbesi, L. Tondi, M. Schiavone, M. Squillace, Palermo: M.G. Carmina, Torino: A. Maina, C. Macchi, E. Gollo, F.M. Comoglio, N. Montali, P. Re, R. Bordese, T. Todros, V. Donvito, W. Grosso Marra, Trieste: G. Sinagra, B. D'Agata Mottolese, M. Bobbo, V. Gesuete, S. Rakar, F. Ramani **JAPAN** - Chiba: K. Niwa **KAZAKHSTAN** - Almaty: D. Mekebekova, A. Mussagaliyeva, T. Lee **KYRGYZSTAN** - Bishkek: E. Mirrakhimov, S. Abilova, E. Bektasheva, K. Neronova, O. Lunegova **LITHUANIA** - Kaunas: R. Žaliūnas, R. Jonkaitienė, J. Petrauskaitė, Vilnius: A. Laucevicius, D. Jancauskaite, L. Lauciuviene, L. Gumbiene, L. Lankutiene, S. Glaveckaite, M. Laukyte, S. Solovjova, V. Rudiene **MALAYSIA** - Kuala Lumpur: K.H. Chee, C.C-W. Yim, H.L. Ang, R. Kuppusamy, T. Watson **MALTA** - Birkirkara: M. Caruana **NORWAY** - Oslo: M-E. Estensen **PAKISTAN** - Rawalpindi: M.G.A. Mahmood Kayani, R. Munir **POLAND** - Bialystok: A. Tomaszuk-Kazberuk, B. Sobkowicz, J. Przepiesc, Krakow: A. Lesniak-Sobelga, L. Tomkiewicz-Pajak, M. Komar, M. Olszowska, P. Podolec, S. Wisniowska-Smialek, Lodz: M. Lelonek, U. Faflik, A. Cichocka-Radwan, Poznan: K. Plaskota, O. Trojnarska **PORTUGAL** - Coimbra: N. Guerra, Lisboa: L. de Sousa, Porto: C. Cruz, V. Ribeiro **REPUBLIC OF MACEDONIA** - Skopje: S. Jovanova **ROMANIA** - Bucharest: V. Petrescu, R. Jurcut, C. Ginghina, I. Mircea Coman, M. Musteata **RUSSIA** - Belgorod: O. Osipova, T. Golivets, I. Khamnagadaev, O. Golovchenko, A. Nagibina, I. Ropatko, Izhevsk: I.R. Gaisin, L. Valeryevna Shilina, Moscow: N. Sharashkina, Saint-

Ramlakhan KP, *et al. Heart* 2021; 107:290–298. doi: 10.1136/heartjnl-2020-317513

Petersburg: E. Shlyakhto, O. Irtyuga, O. Moiseeva, E. Karelkina, I. Zazerskaya, A. Kozlenok, I. Sukhova **SERBIA** - Belgrade: L. Jovovic **SLOVENIA** - Ljubljana: K. Prokšelj, M. Koželj **SOMALILAND** - Hargeisa: A.O. Askar, A.A. Abdilaahi, M.H. Mohamed, A.M. Dirir **SOUTH AFRICA** - Cape Town: K. Sliwa, Houghton: P. Manga **SPAIN** - Barcelona: A. Pijuan-Domenech, L. Galian-Gay, P. Tornos, M.T. Subirana, M. T. Subirana , Bilbao: N. Murga, Madrid: J. M. Oliver, B. Garcia-Aranda Dominguez, I. Hernandez Gonzalez, J.F. Delgado Jimenez, P. Escribano Subias **SUDAN** - Khartoum: A. Elbushi, A. Suliman, K. Jazzar, M. Murtada, N. Ahamed **SWEDEN** - Göteborg: M. Dellborg, E. Furenas, M. Jinesjo, K. Skoglund, P. Eriksson, T. Gilljam, Lund: U. Thilen **SWITZERLAND** - Basel: D. Tobler, Bern: K. Wustmann, F. Schwitz, M. Schwerzmann, Lausanne: T. Rutz, J. Bouchardy, Zurich: M. Greutmann, B.M. Santos Lopes, L. Meier, M. Arrigo **THE NETHERLANDS** - Amsterdam: K. de Boer, T. Konings, Enschede: E. Wajon, L.J. Wagenaar, Geldrop: P. Polak, Groningen: E.PG. Pieper, Rotterdam: J. Roos-Hesselink, L. Baris, I. van Hagen, H. Duvekot, J.M.J. Cornette, The Hague: C. De Groot, Utrecht: C. van Oppen **TURKEY** - Istanbul: L. Sarac, O. Batukan Esen, S. Catirli Enar **UGANDA** - Kampala: C. Mondo, P. Ingabire, B. Nalwanga, T. Semu **UNITED ARAB EMIRATES** - Abu Dhabi: B.T. Salih, W.A.R. Almahmeed, S. Wani, F.S. Mohamed Farook, Al Ain, F. Gerges, A.M. Komaranchath, F. Al bakshi, Dubai: A. Al Mulla, A.H. Yusufali, E.I. Al Hatou, N. Bazargani, F. Hussain **UNITED KINGDOM** - Birmingham: L. Hudsmith, P. Thompson, S. Thorne, S. Bowater, Buckinghamshire: A. Money-Kyrle, P. Clifford, P. Ramrakha, S. Firoozan, J. Chaplin, N. Bowers, Coventry: D. Adamson, London: F. Schroeder, R. Wendler, S. Hammond, P. Nihoyannopoulos, Norwich Norfolk: R. Hall, L. Freeman, Southampton: G. Veldtman, J. Kerr, L. Tellett **UNITED STATES** - Boston: N. Scott, A.B. Bhatt, D. DeFaria Yeh, M.A. Youniss, M. Wood, A.A. Sarma, S. Tsiaras, A. Stefanescu, J.M. Duran, L. Stone, Cleveland: D.S. Majdalany, J. Chapa, Detroit: K. Chintala,

P. Gupta , Hershey, PA: J. Botti, J. Ting, W. R. Davidson, Lexington, Kentucky: G. Wells, D. Sparks, Mineola, NY: V. Paruchuri, K. Marzo, D. Patel, Minneapolis: W. Wagner, S.N. Ahanya, L. Colicchia, T. Jentink, K. Han, M. Loichinger, M. Parker, W. Wagner, C. Longtin, Omaha: A. Yetman, K. Erickson, J. Cramer, S. Tsai, B. Fletcher, S. Warta, Phoenix: C. Cohen, C. Lindblade, R. Puntel, K. Nagaran, N. Croft, Seattle: M. Gurvitz, C. Otto, Stanford, CA: C. Talluto, D. Murphy, M. G. Perlroth
